# A follow-up report on the published paper Social and clinical impact of COVID-19 on patients with fibrodysplasia ossificans progressiva

**DOI:** 10.1186/s13023-023-02638-0

**Published:** 2023-03-20

**Authors:** Hayley Wallace, Rhonda H. Lee, Edward C. Hsiao

**Affiliations:** 1 Division of Endocrinology and Metabolism, The UCSF Metabolic Bone Clinic, University of California-San Francisco, 513 Parnassus Ave., HSE901G, San Francisco, CA 94143-0794 USA; 2 The Institute for Human Genetics, the Program in Craniofacial Biology, and the Robert L. Kroc Chair in Rheumatic and Connective Tissue Diseases III, University of California, San Francisco, San Francisco, CA USA

**Keywords:** Fibrodysplasia Ossificans Progressiva (FOP), COVID-19, SARS-CoV-2, Vaccine, Heterotopic Ossification (HO)

## Abstract

Fibrodysplasia ossificans progressiva (FOP) is a rare genetic disorder associated with increased immune activity and severe, progressive heterotopic ossification. We previously described a cohort of 32 patients with FOP who were either exposed to SARS-CoV-2 or received a COVID-19 vaccine^1^ and showed that these patients did not develop heterotopic ossification after COVID-19 vaccination. Here, we present additional clinical data from new subjects and additional long-term follow-up from the first cohort. We enrolled 15 new subjects between August 24th, 2021 and May 17th, 2022 and collected additional self-reported outcomes. The larger cohort with 47 individuals encompassing 49 events showed that patients with FOP exhibited no additional change in FOP disease activity or flare activity resulting from COVID-19 infection or after receipt of a SARS-CoV-2 vaccine. Thus, although any vaccination carries a risk of inducing heterotopic ossification in patients with FOP, our results show that patients with FOP who choose to receive a COVID-19 vaccination may be able to tolerate the procedure without a high risk of heterotopic ossification when following the published guidelines.

## Dear Editor,

Fibrodysplasia ossificans progressiva (FOP) is a rare genetic disorder associated with increased immune activity and severe, progressive heterotopic ossification (HO). Patients with FOP develop significant respiratory compromise from HO affecting their chest wall, as well as cardiopulmonary dysfunction and thoracic syndrome. These physical changes put patients with FOP at higher risk of medical complications from respiratory infections. In addition, the pro-inflammatory nature of FOP produces an increased susceptibility to HO from traumatic stimuli, even minor events, which means any immunization carries significant risk of HO formation. Thus, understanding the risks of COVID-19 and vaccination are critical for helping patients with FOP make informed medical decisions about vaccination.

We previously described 32 subjects with FOP who tested positive for SARS-CoV-2, had a high-risk exposure, or received COVID-19 vaccination that were recruited between July 14th, 2020 and August 23rd, 2021 in a study approved by the UCSF Institutional Review Board [1]. We showed that patients with FOP who received the COVID-19 vaccination and followed guidelines from the International Clinical Council (ICC) on FOP [2] generally tolerated the vaccine well. Subjects who tested positive for the SARS-CoV-2 virus showed no major complications for increased FOP disease activity. Only one patient among 15 subjects who received the COVID-19 vaccine experienced a flare at the injection site.

Since the initial publication, we identified 15 additional new subjects and performed follow-up assessments on prior subjects until May 17th, 2022. Subjects were enrolled if they tested positive for COVID-19 and/or if they received one of the COVID-19 vaccines. We did not include cases of high-risk exposure lacking a confirmed positive diagnosis. No data was collected on social or behavioral characteristics in this extension study.

## Data collection methods

Demographics including age, sex, and country of origin were collected immediately following consent. The same two patient-reported outcomes questionnaires administered during the data collection of the initial phase^1^ were used during the second phase. The questionnaires were tailored for the respective recipient’s experience, either to obtain information on the COVID-19 infection or close contact exposure, or to obtain information about the vaccine received. Outreach was conducted on participants enrolled in the first phase to learn if those who contracted COVID-19 or experienced a high-risk exposure later received a vaccine or if those who received a vaccine later obtained a booster shot. The new clinical data were added to the data collected during the first timeframe for updated analysis. The total data were converted to case events because some subjects had more than one infection.

## Results

**Fig. 1:**
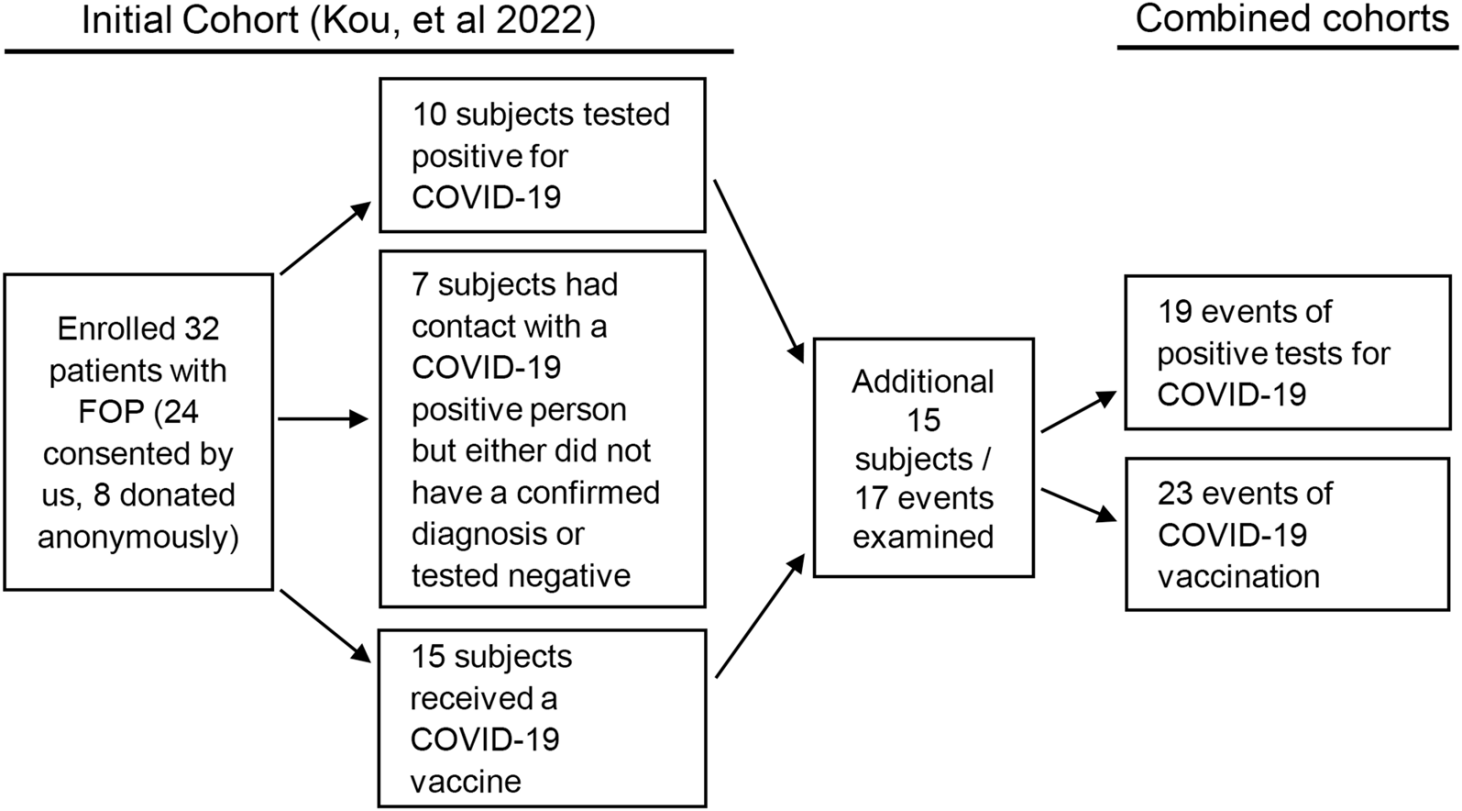
COVID-19 and SARS-CoV-2 vaccine study subjects flow chart from published paper to follow-up period

Our initial report described 32 subjects, 10 of whom tested positive for COVID-19, seven reported close contact with a COVID-19 positive case but did not have a confirmed diagnosis themselves, and 15 received at least one dose of a COVID-19 vaccine (Fig. 1). Fifteen new subjects were enrolled during the follow-up period with five subjects from the first enrollment cohort reporting new information. Figure 1 shows an updated flow-chart from our published paper showing updated numbers for total subjects and events observed, broken down by COVID-19 infection and vaccine attainment. Tables 1, 2, and 3 are updated with the pooled data obtained during both observation timeframes for re-analysis. Table 4 shows a breakdown of the vaccine manufacturers and injection type.

**Table 1:** COVID-19 (+) subjects and vaccination subjects Demographics

Characteristic	Pooled COVID-19 ( +) study subjects (N = 19)	Pooled vaccination study subjects (N = 23)
*Current age (years)*		
Mean	27.5	34
Range	8–65	13–64
*Sex*		
Male	12	10
Female	7	13
*Country/region*		
Argentina	3	1
Brazil	1	0
Canada	0	1
Chile	0	1
France	0	1
Serbia	1	0
Sweden	1	0
Switzerland	0	1
Turkey	1	0
United Kingdom	1	2
USA	11	16

**Table 2:** COVID-19 (+) subject-reported symptoms/outcomes (*N* = 19)

Characteristic	Number of subjects	Frequency (%)
Weak/fatigued	13	68.4
Loss of sense of smell or taste	10	52.6
Cough	13	68.4
Fever	9	47.4
Sore throat	8	42.1
Headache	3	15.8
Muscle aches	5	26.3
Diarrhea	4	21
Difficulty breathing	3	15.8
Flaring	2	10.5
Eye redness	2	10.5
Chills/shakes	4	21
Hospitalization	3	15.8
Runny/congested nose	5	26.3

**Table 3:** COVID-19 vaccine subject-reported side effects/outcomes (*N* = 23)

Characteristic	Number of subjects	Frequency (%)
Pain/soreness	19	82.6
Tiredness/fatigue	10	43.5
Swelling	5	21.7
Headaches	7	30.4
Fever	1	4.3
Flare	1	4.3
Chills	2	8.7
Hospitalization	0	0
HO formation	0	0

**Table 4:** COVID-19 vaccine type and injection method (*N* = 23)

Manufacturer	Number of subjects	Frequency (%)
Pfizer	16	69.5
Johnson & Johnson	3	13
Moderna	3	13
CoronaVac	1	4.3

Injection location	Number of subjects	Frequency (%)
Intramuscular	20	86.9
Subcutaneous	1	4.3
Mixed	1^1^	4.3
Not reported	1	4.3

In the new aggregate cohort of 47 subjects, there were 19 events of positive tests for COVID-19 and 23 events of receiving at least one dose of a vaccine. Doses given as part of a multi-dose treatment protocol were counted together as one vaccination event. The mean age for subjects who tested positive for COVID-19 was 27.5 years old while the mean age for participants who received at least one vaccine dose was 34 years old. More males than females reported a positive viral diagnosis whereas more females than males reported obtaining a vaccine. Country of origin data remained consistent with the initial paper’s cohort, with the United States still ranking the highest and lower representation from Europe and Latin America. This report bias may be driven by several factors, including that the study was based in the United States, that vaccine access was highly variable across different countries, and that the largest proportion of participants in the IFOPA registry is from North America [3]

Patient reported outcomes by subjects who tested positive for COVID-19 did not significantly change with the addition of new enrollees. The most common symptoms were weakness/fatigue and cough, both with a frequency of 68.4%. The following most common symptoms were loss of sense of taste and smell with 52.6% frequency and fever with 47.4% frequency. Only three participants were hospitalized for their symptoms and no subjects reported flare activity in the weeks following infection.

Patient reported outcomes by subjects who received a standard vaccine course per the manufacturer or vaccine plus booster shot also did not change significantly with the addition of new enrollees and follow-up among subjects enrolled during the first cohort. Pain/soreness at the injection site was the most common symptom with 82.6% frequency followed by tiredness/fatigue with 43.5% frequency. Only one subject reported a flare in the first two weeks following the injection (from the initial cohort) and no subjects reported heterotopic ossification (HO).

Most subjects received the Pfizer vaccine compared to Moderna, Johnson & Johnson, and CoronaVac. Nearly all subjects received their injections intramuscularly. Only three subjects deviated from this: one did not report data, one received the vaccine subcutaneously, and one subject mixed their injection methods receiving the first injection of a two-dose vaccine subcutaneously but intramuscularly for the second injection.

## Discussion

During the total of 22 months of observation, we found that vaccination of patients with FOP with the COVID-19 vaccines could be well tolerated, using the guidelines published in our original manuscript [1] and on the ICCFOP.org website [2]. ICC FOP guidelines for COVID-19 Vaccine Injections included taking the vaccine through its intended route, taking the vaccine at a location that is already fused, avoiding vaccination sites exposed to pressure (such as the buttocks), being flare-free for at least 2 weeks prior, using the smallest diameter needle available, and taking ibuprofen or acetaminophen before the vaccination and for 48 h after the injection. The risks of a trauma-related flare near an injection site and/or HO formation remain major concerns for all patients with FOP. Thus, our findings that the COVID-19 vaccination can be tolerated in patients with FOP should not be extended to other intramuscularly administered vaccinations. In addition, there has been at least two case reports of patients with FOP or suspected FOP having post-COVID-19 exacerbation of flare up and HO formation activity [6, 7]. There are reported cases of higher prevalence of HO in patients with ARDS due to prolonged immobilization [8]. Some vaccinated patients with FOP have also shown strong SARS-CoV-2 specific humoral responses, which did not significantly differ from responses of healthy individuals [9]. In addition, COVID-19-related HO may show less severity compared to other types of HO [10]. Together, these long-term data may be useful for patients with FOP as they work with their medical team to weigh the relevant risks and benefits of a COVID-19 vaccination.

## Conclusion

Due to the propensity for post-traumatic flare activity and progressive HO formation characteristic of FOP, we had originally hypothesized that COVID-19 infection and vaccination may have disproportionately negative impacts on people with FOP. Our observations on a small cohort suggest that patients with FOP have similar risks as the general population to COVID-19 infection, and that patients with FOP may be able to tolerate COVID-19 vaccination when treated following a consensus protocol to mitigate flare risk [1]. Because other vaccinations and viral infections are known [4–7] to be associated with HO formation in patients with FOP, this information should be used to guide individual discussions about risks and benefits for vaccination within the FOP community.

## Data Availability

The data that supports the findings of this study are available from the corresponding author upon reasonable request.

## Abbreviations

FOP: Fibrodysplasia Ossificans Progressiva
HO: Heterotopic Ossification
COVID-19: Coronavirus Disease
SARS-CoV-2: Severe Acute Respiratory Syndrome coronavirus 2

## References

[1] Kou S, Kile S, Kambampati SS, Brady EC, Wallace H, De Sousa CM (2022). Social and clinical impact of COVID-19 on patients with fibrodysplasia ossificans progressive. Orphanet J Rare Dis.

[2] International Clinical Council (ICC) on Fibrodysplasia Ossificans Progressiva (FOP). 2022. *Guidelines—International Clinical Council (ICC) on Fibrodysplasia Ossificans Progressiva (FOP)*. https://www.iccfop.org/guidelines/. Accessed 23 September 2022.

[3] Liljesthröm M, Pignolo R, Kaplan F (2020). Epidemiology of the Global Fibrodysplasia Ossificans Progressiva (FOP) community. J Rare Dis Res Treat.

[4] Lanchoney T, Cohen R, Rocke D, Zasloff M, Kaplan F (1995). Permanent heterotopic ossification at the injection site after diphtheria-tetanus-pertussis immunizations in children who have fibrodysplasia ossificans progressiva. J Pediatr.

[5] Scarlett R, Rocke D, Kantanie S, Patel J, Shore E, Kaplan F (2004). Influenza-like viral illnesses and flare-ups of fibrodysplasia ossificans progressiva. Clin Orthop Relat Res.

[6] Grgurevic L, Novak R, Hrkac S, Salai G, Grazio S (2021). Post-COVID-19 exacerbation of fibrodysplasia ossificans progressiva with multiple flare-ups and extensive heterotopic ossification in a 45-year-old female patient. Rheumatol Int.

[7] Brance ML, Cóccaro NM, Casalongue AN, Durán A, Brun LR (2022). Extensive progressive heterotopic ossification post-covid-19 in a man. Bone.

[8] Stoira E, Elzi L, Puligheddu C, Garibaldi R, Voinea C, Chiesa AF (2021). High prevalence of heterotopic ossification in critically ill patients with severe COVID-19. Clin Microbiol Infect.

[9] Smetanova J, Milota T, Rataj M, Hurnakova J, Zelena H, Horvath R (2022). SARS-COV-2-specific humoral and cellular immune responses to BNT162B2 vaccine in fibrodysplasia ossificans progressiva patients. Front Immunol.

[10] Mezghani S, Salga M, Tordjman M, Amar R, Carlier R-Y, Chiche L (2022). Heterotopic ossification and COVID 19: imaging analysis of ten consecutive cases. Eur J Radiol.

